# Panoramic on Epigenetics in Coronary Artery Disease and the Approach of Personalized Medicine

**DOI:** 10.3390/biomedicines11102864

**Published:** 2023-10-23

**Authors:** Marcello Bergonzini, Francesco Loreni, Antonio Lio, Marco Russo, Guglielmo Saitto, Antonio Cammardella, Francesco Irace, Corrado Tramontin, Massimo Chello, Mario Lusini, Antonio Nenna, Chiara Ferrisi, Federico Ranocchi, Francesco Musumeci

**Affiliations:** 1 Department of Cardiac Surgery and Heart Transplantation, San Camillo Forlanini Hospital, 00152 Rome, Italy; 2 Cardiac Surgery, Università Campus Bio-Medico di Roma, 00128 Rome, Italy

**Keywords:** cardiovascular diseases, clinical epigenetics, biomarkers, drug targets, DNA methylation, histone modification

## Abstract

Epigenetic modifications play a fundamental role in the progression of coronary artery disease (CAD). This panoramic review aims to provide an overview of the current understanding of the epigenetic mechanisms involved in CAD pathogenesis and highlights the potential implications for personalized medicine approaches. Epigenetics is the study of heritable changes that do not influence alterations in the DNA sequence of the genome. It has been shown that epigenetic processes, including DNA/histone methylation, acetylation, and phosphorylation, play an important role. Additionally, miRNAs, lncRNAs, and circRNAs are also involved in epigenetics, regulating gene expression patterns in response to various environmental factors and lifestyle choices. In the context of CAD, epigenetic alterations contribute to the dysregulation of genes involved in inflammation, oxidative stress, lipid metabolism, and vascular function. These epigenetic changes can occur during early developmental stages and persist throughout life, predisposing individuals to an increased risk of CAD. Furthermore, in recent years, the concept of personalized medicine has gained significant attention. Personalized medicine aims to tailor medical interventions based on an individual’s unique genetic, epigenetic, environmental, and lifestyle factors. In the context of CAD, understanding the interplay between genetic variants and epigenetic modifications holds promise for the development of more precise diagnostic tools, risk stratification models, and targeted therapies. This review summarizes the current knowledge of epigenetic mechanisms in CAD and discusses the fundamental principles of personalized medicine.

## 1. Introduction

Epigenetics is described as the changes that impact variations in phenotype without altering the DNA sequence. These changes occur through covalent modifications in chromatin components, DNA bases, and the expression of non-coding RNA species, influencing gene expression patterns. Ultimately, they play a crucial role in maintaining cellular identity, which is vital for normal development and the occurrence of diseases. Over the past two decades, epigenetics has gained significant attention in the field of precision medicine, as epigenetic changes have been linked to various pathological conditions, including both malignant and non-malignant disorders, particularly those related to lifestyle. These changes are often influenced by modifications in the cellular microenvironment, which, in turn, are determined by various modifiable and non-modifiable factors.

Considering these facts, it is indisputable that epigenetics plays a role in the development of coronary artery disease (CAD). Moreover, delving into the realm of epigenetics across various diseases has opened doors to the creation of novel therapeutic solutions. Acute myocardial infarction (AMI) stands as a prominent global cause of mortality. The impact of epigenetic shifts in individuals with CAD is a relatively fresh concept that holds captivating implications. Primary pathophysiological changes leading to CAD include endothelial dysfunction, imbalanced cholesterol metabolism, and the formation of atherosclerotic plaques.

These modifications, combined with risk factors associated with obesity such as diabetes (DM II or I), SAH (systemic arterial hypertension), and insulin resistance, are connected to environmental factors that contribute to the increased occurrence of CAD. Epigenetic modifications manifest in various cell types, including vascular smooth muscle cells (VSMC), endothelial cells (EC), and macrophages, subsequent to the formation of atherosclerotic plaques. Atherosclerotic plaque development, which constitutes the primary pathological change leading to CAD, is associated with inflammation, cholesterol metabolism, and homocysteine homeostasis. The body of literature on the impact of epigenetics on CAD is expanding rapidly, forming a foundation for delving into the underlying epigenetic mechanisms of the disease. We will delve into the most recent news concerning epigenetic alterations that occur during the adult stages of CAD. Furthermore, we will explore the interplay between an individual’s epigenetics and their environment in instigating and advancing CAD. Additionally, we will investigate epigenetics in personalized medicine, providing readers with a comprehensive overview [1,2,3,4].

## 2. Coronary Artery Disease

Atherosclerosis represents a persistent inflammatory state characterized by the thickening of blood vessel walls, resulting in the blockage of these vessels. This pathological process forms the foundation for various systemic disorders, including CAD and peripheral artery disease. The atherosclerotic plaque is essentially a buildup of lipids, T lymphocytes, macrophages, and fibrous tissue within the walls of arteries. This process is in accordance with the “response to injury” hypothesis, which commences with endothelial dysfunction as the initial event in a series of pathological transformations that typify this enduring inflammatory condition. Notably, this dysfunction emerges prior to any structural changes in the arterial walls. The oxidation of low-density lipoprotein (LDL) cholesterol within the subendothelial space plays a pivotal role, leading to the expression of cell adhesion molecules. This, in turn, causes monocytes to adhere to and migrate into the subendothelial region. Inside the intima, these monocytes differentiate into macrophages, which subsequently ingest oxidized LDL via scavenger receptors. This sequence of events promotes the formation of foam cells and triggers the release of pro-inflammatory cytokines, including interleukins and tumor necrosis factor. The formation of an atherosclerotic lesion, known as a fatty streak, represents the final stage of this process. Atherosclerotic plaque accumulation leads to vascular remodeling, luminal obstruction, abnormal blood flow, and reduced oxygen and nutrient supply to the myocardium. These changes weaken or obstruct the normal blood flow in the coronary arteries, resulting in myocardial ischemia. In this review, we will discuss the significant role of epigenetic changes in the development of coronary artery disease (CAD).

Risk factors for CAD can be categorized into demographic, biochemical, lifestyle, and genetic factors. Demographic factors encompass age and male gender, while biochemical factors relate to elevated cholesterol levels. Lifestyle factors encompass sedentary work habits, high levels of stress and depression, imbalanced dietary habits, socioeconomic status, and tobacco smoking or alcohol abuse. Genetic factors include a positive family history of CAD and inherited lipid metabolism disorders [5,6,7,8,9,10,11,12,13].

Liu F. et al. [14] findings indicate that the *m.5178C* > A variant is linked to elevated levels of high-density lipoprotein cholesterol (HDL-C), standardized mean difference (SMD) = 0.12, 95%, confidence interval (CI) = 0.06–0.17, *p* < 0.001], and total cholesterol (TC) (SMD = 0.08, 95% CI = 0.02–0.14, *p* = 0.01). When we examined specific populations, we found that the *m.5178C* > A variant was associated with increased HDL-C levels in both Japanese (SMD = 0.09, 95% CI = 0.01–0.17, *p* = 0.03) and Chinese (SMD = 0.13, 95% CI = 0.07–0.20, *p* < 0.001) groups. However, the connection between the *m.5178C >* A variant and decreased levels of low-density lipoprotein cholesterol (LDL-C) was only observed in the Japanese population (SMD = −0.11, 95% CI = −0.22 to 0.00, *p* = 0.04). The presence of the *m.5178C* > A variant in Japanese populations was linked to elevated levels of HDL-C (high-density lipoprotein cholesterol) and reduced levels of LDL-C (low-density lipoprotein cholesterol). These findings suggest that this genetic variation may play a role in reducing the risk of coronary artery disease (CAD) and contributing to the longer lifespan observed among Japanese individuals. The mechanisms that explain the connection between the *m.5178C* > A variant and lipid levels are not fully understood at this time. Nonetheless, one hypothesis that could be put forward to clarify our results is related to its potential role in safeguarding against oxidative stress.

**Figure 1:**
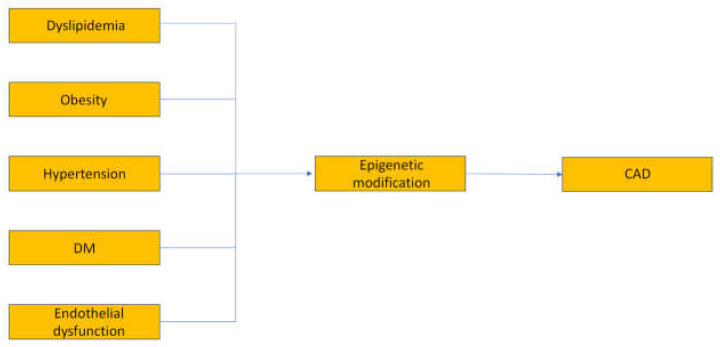
Increased rick of developing CAD.

Age plays a fundamental role in CAD development, as advancing age is an independent risk factor. Men have a higher mortality rate from CAD compared to pre-menopausal women. However, after menopause, the incidence of CAD in women becomes similar to that in men of the same age. Unhealthy lifestyle choices, such as obesity, are associated with epigenetic alterations, including histone modifications and DNA methylation, which contribute to the development of CAD. Tobacco smoking has been linked to DNA methylation changes in atherosclerotic carotid lesions. Studies also indicate that specific DNA methylation patterns are found in CAD patients who smoke. However, quitting smoking can reduce the risk of developing CAD and related diseases. Chronic alcohol consumption has also been shown to affect DNA methylation, leading to impaired lipid metabolism and CAD. Furthermore, environmental exposure during early childhood, particularly in the prenatal period, can induce metabolic and physiological changes through epigenetic modifications. This may increase the susceptibility to chronic diseases like diabetes, obesity, cardiovascular events, and cancer. The reversible nature of epigenetic changes makes them a promising target for therapeutic interventions [15,16,17,18,19,20,21,22,23,24] (Figure 1).

## 3. Covalent Modifications

### 3.1. DNA Methylation

A pre-transcriptional covalent modification is widely studied and considered the primary epigenetic modification in mammals. It involves the addition of a methyl group (-CH3) to the fifth carbon of cytosine within CpG dinucleotide regions. This process is facilitated by specific enzymes known as DNA methyltransferases (DNMTs). DNA methylation plays a crucial role in various biological activities, including gene expression, cellular function, aging, and the development of both malignant and non-malignant conditions such as CAD. In the human genome, approximately 70% to 80% of CpG dinucleotides are methylated, forming clusters known as CpG islands that function as regulatory units for gene expression by influencing the binding of transcription factors. Promoter regions typically possess a greater abundance of CpG dinucleotides in comparison to intronic and exonic regions. Elevated methylation levels in promoter regions of somatic cells can impede gene activity, whereas unmethylated promoters are linked to active gene expression [25,26,27,28].

Lipid metabolism plays a critical role in the development of atherosclerosis and is influenced by a combination of genetic and environmental factors. Specific genes, such as insulin-like growth factor 2 (IGF2) and leptin, have shown instances of hypermethylation risk factors for CAD, like obesity. In contrast, the hypomethylation of the ATP-binding cassette transporter G1 (ABCG1) gene and hypermethylation of carnitine palmitoyltransferase 1A (CPT1A) have been correlated with postprandial lipemia. Distinct methylation patterns are observed in genes implicated in CAD pathogenesis and lipid regulation [29,30,31,32,33,34,35].

The development of coronary artery disease (CAD) is shaped by a combination of environmental and lifestyle factors that contribute to alterations in the epigenome. These epigenetic modifications can occur even during fetal development, which is a critical period that can program an individual’s disease risk in adulthood. Exposure to smoking, environmental toxins, and pollution during early and mid-life stages can induce DNA methylation, laying the foundation for non-genetic mechanisms through which CAD may develop later in life. Furthermore, dietary factors such as a low-protein diet and deficiencies in vitamin B12, folic acid, and methionine during pregnancy, as well as a low-fat diet rich in flavonoids, can affect the methylation patterns of specific genes involved in atherogenesis. Numerous studies have highlighted the significance of DNA methylation changes in regulating various biological processes associated with CAD. For example, mice with hypomethylated DNA display increased expression of inflammatory markers, leading to the formation of fatty streaks in the aorta. In mice predisposed to CAD due to the absence of ApoE, specific DNA methylation modifications have been detected in both blood leukocytes and the aorta even before the onset of vascular lesions. These modifications play a role in inflammation and the formation of atherosclerotic plaques. While global hypomethylation has been observed in DNA derived from atherosclerotic tissues, genes associated with CAD more commonly exhibit hypermethylation in promoter regions. These differences may be attributed to the presence of epigenetically/genetically distinct cell populations within the atherosclerotic tissue. Additionally, different methylation patterns may be associated with heterogeneous cell types within an individual’s tissue. However, hypermethylation seems to have a more significant impact on the development of CAD [36,37,38,39,40,41,42,43,44,45,46,47,48].

An alternative approach to examining methylation patterns involves analyzing the methylation of CpG dinucleotides in target genes. Several reports from ongoing studies focus on the pathophysiology of atherogenesis. Alterations in the methylation status of particular genes of interest can influence functional pathways associated with atherogenesis. These pathways encompass LDL metabolism, lipid homeostasis, cholesterol biosynthesis, homocysteine metabolism, endothelial dysfunction, and inflammation. As a result, the examination of methylation patterns in candidate genes associated with coronary artery disease (CAD) using advanced techniques serves to confirm the involvement of new genes discovered through comprehensive methylation studies. This paves the way for potential targeted therapies and the development of prognostic markers once these findings are validated [49,50,51,52,53,54] (Table 1).

### 3.2. Histon Modifications

Histon modifications involve alterations in alkaline proteins called histones. Histones are divided into five categories: H1/H5, H2A, H2B, H3, and H4. They serve as fundamental units of chromatin, but their structure is highly dynamic and can be rapidly modified in response to external signals. Various post-translational modifications, such as acetylation, methylation, and phosphorylation, occur on histone molecules, affecting chromatin structure, organization, and function. These modifications play a crucial role in transcriptional regulation, gene expression, DNA repair, and overall cellular function. In the context of CAD, extensive research has focused on histone methylation and demethylation, exploring their implications and potential involvement in the disease [55,56,57,58,59].

The methylation of histones is intricately associated with the activation or deactivation of particular genes; the modified segment of the histone determines their activation or silencing. The model of histone methylation was first identified in the 1960s, and the discovery of lysine methyltransferase (KMT) family enzymes provided insight into the methylation process. Histone methylation was initially considered a post-translational modification that was stable and irreversible, until the detection of the first histone lysine demethylase (KDM) enzymes. Histone methylation can occur on both arginine and lysine residues, with documented methylation residues in histones H3 and H4. The methylation of lysine residues can occur in different degrees, such as mono(me1), di(me2), or trimethyl (me3) groups. Histone lysine methyltransferases act as catalysts, transferring methyl groups from S-adenosylmethionine (SAM) to lysine residues on the histone tails. The discovery of lysine demethylase enzymes has challenged the notion of histone methylation as an irreversible and stable modification. Two critical classes of histone lysine dimethyl enzymes have been identified, including lysine-specific demethylase 1 (LSD1) and proteins containing the JmjC domain, which participate in histone demethylation using cofactors such as flavin adenine dinucleotide and Fe(II) with alpha-ketoglutarate.

While arginine demethylase enzymes have undergone extensive research, histone lysine demethylation takes on a primary role. Many studies have presented evidence of the link between histone methylation and the development of atherosclerotic plaques. For instance, atherosclerotic plaques have been shown to exhibit reduced levels of H3K9me2 and H3K27me2, while H3K4me2 levels were found to be elevated in vascular smooth muscle cells (VSMCs). Additionally, histone methyltransferases MLL2 and G9a were increased in the advanced stages of atherosclerosis. Furthermore, vessels with advanced atherosclerotic plaques displayed a decrease in global H3K27me3 levels, implying a significant connection between H3K9 and H3K27 demethylation and the formation of plaques. The methylation of histone H3K4 has been associated with the initiation and advancement of CAD, while the elevated demethylation of H3K27 is observed in the advanced stages of CAD. Histone proteins play a crucial role due to their close association with DNA, influencing various regulatory processes. Nevertheless, the precise role of histones in CAD is still being uncovered, and additional research is necessary to establish specific models [60,61,62,63,64,65,66,67,68,69,70]. Histone acetylation, a discovery dating back around 50 years, is directly associated with the activation or deactivation of specific genes. The modified portion of the histone determines whether a gene is turned on or silenced. In the 1960s, the concept of histone methylation was first identified, although the enzyme responsible for methylation remained unknown until the discovery of lysine methyltransferase (KMT) enzymes. Histone methylation was once considered the primary post-translational modification until the identification of the first histone lysine demethylase (KDM). Methylation can take place on both arginine and lysine residues in histones H3 and H4. Histone lysine methyltransferases serve as catalysts, facilitating the transfer of a methyl group from S-adenosylmethionine (SAM) to amino groups on lysine residues located in the histone tails. The discovery of lysine demethylase enzymes has challenged the notion that histone methylation is irreversible and stable. Two critical classes of histone lysine demethylases have been identified, with lysine-specific demethylases (LSDs) reported to use flavin adenine dinucleotide as a cofactor. LSD1, the most studied LSD, interacts with protein complexes such as CoREST (corepressor of RE1-silencing transcription factor) and other protein complexes, catalyzing reversible methylation of H3K4 and H3K9. Recent studies have highlighted that JmjC domain-containing proteins constitute the largest class of histone demethylases. These proteins serve as catalysts and play a pivotal role in histone demethylation, utilizing Fe(II) and alpha-ketoglutarate as cofactors. While arginine demethylases have been extensively investigated, histone lysine demethylation remains primarily associated with the regulation of gene transcription. Numerous studies have provided evidence for the connection between histone methylation and atherosclerotic plaque formation. In 2015, Greibel et al. established that H3K9me2 and H3K27me2 exhibited significant reductions within atherosclerotic plaques. Conversely, they observed elevated levels of H3K4me2 in atherosclerotic carotid arteries when compared to healthy arteries.

Concurrently, results from immunohistochemistry have revealed an elevation in H3K4me2 levels within vascular smooth muscle cells, while H3K9me2 has shown a decrease. Similarly, both H3K9me2 and H3K27me2 levels were diminished in inflammatory cells. Interestingly, the expression of the corresponding histone methyltransferases MLL2 and G9a was found to be higher in the advanced stages of atherosclerosis when compared to the early stages. Moreover, in 2015, Wierda and colleagues noted that there were reduced global levels of H3K27me3 in vessels with advanced plaques. Significantly, this outcome did not affect the expression of the histone methyltransferase EZH2 or the demethylase JMJD3, suggesting that H3K9 and H3K27 demethylation were closely associated with the formation of atherosclerotic plaques. H3K4 histone methylation has been linked to the initiation and progression of coronary atherosclerosis. However, increased H3K27 demethylation has been observed in the advanced stage of CAD. The considerable influence of histone proteins stems from their intimate interaction with DNA, rendering them indispensable in various regulatory processes. Consequently, histones serve as critical components that exert an exceptionally significant role in both the onset and advancement of the CAD phenotype. Nevertheless, their exact roles are diverse, and a specific model has yet to be established [71,72,73,74,75,76,77,78,79].

The phosphorylation of histone proteins occurs when hydroxyl groups of serine, threonine, and tyrosine residues are modified. Kinases act as catalysts, adding a phosphate group from ATP, while phosphatases remove the phosphate group. This addition of a phosphate group introduces a negative charge, which plays a role in chromatin remodeling and gene transcription. However, calcium/calmodulin-dependent protein kinase II δ (CAMKII δ) has been found to have a significant role in the pathogenesis of cardiac hypertrophy. It has been reported that nuclear CAMKII δ facilitates chromatin remodeling by phosphorylating H3Ser10, thereby promoting gene transcription and contributing to coronary diseases [77,80,81,82].

## 4. Non-Coding RNAs

The NIH-led “ENCODE” project has reported that only 3% of the human genome is responsible for protein translation, while 86% is transcribed into ncRNAs. In the past, it was believed that the non-coding regions of the genome had no significant in organism development and were considered “junk DNA”. Recent studies have revealed the crucial role of non-coding regions in gene expression regulation, protein control, and the development of various diseases, including coronary atherosclerosis. These non-coding RNAs serve important regulatory and structural functions. They can be classified into small ncRNAs (less than 200 nucleotides) and long ncRNAs (over 200 nucleotides). Today, ncRNAs are recognized as significant biomarkers for cardiovascular damage and play a part in the pathogenesis of CAD [83,84,85,86,87,88,89,90].

### 4.1. miRNA

The discovery of microRNAs (miRNAs) has revolutionized translational research. These small molecules play a crucial role in regulating gene expression by modulating the activity of multiple genes through various molecular mechanisms. The nucleotide sequences of miRNAs are highly conserved across different species, indicating their important role in gene regulation. In the human genome, approximately 2500 miRNAs have been identified, and they are responsible for regulating around 62% of human genes. Extensive studies have investigated the involvement of miRNAs in CAD, highlighting their significant role in the development and progression of CAD. Several studies have reported the impact of specific miRNAs in CAD. Additionally, miRNAs such as miRNA-1 and miRNA-133a have shown promise as diagnostic and prognostic biomarkers for acute myocardial infarction (AMI), surpassing the efficacy of traditional cardiac troponin markers [91,92,93] (Table 2).

### 4.2. Long Non-Coding RNA (lncRNA)

In 2002, Okazaki and his team made the groundbreaking discovery of long non-coding RNAs (>200 bp) during extensive sequencing in a mouse model. Subsequent research has demonstrated that these long non-coding RNAs (lncRNAs) can be expressed either in the cytoplasm or in the nucleus. Due to their remarkable stability, lncRNAs have become intriguing entities and essential subjects of research. They can exist in extracellular fluid, such as plasma, with exceptional stability, exhibiting complex behavior in various pathological conditions. Packaged within exosomes or extracellular vesicles, these lncRNAs can be released by dying or apoptotic cells and enter the bloodstream. The packaging in exosomes enhances their stability and prolongs their half-life. Furthermore, lncRNAs form stable complexes with RNA-binding proteins, protecting them from degradation by RNases. Their extended half-life and enhanced stability make them easily detectable and potential non-invasive biomarkers for diagnosis and prognosis. Similar to miRNAs, lncRNAs do not encode proteins and exhibit mRNA-like structures. Among the various types of lncRNAs, long intergenic non-coding RNAs (lincRNAs) have been extensively studied and found to have significant implications in various diseases, including CAD immune disorders, and different malignancies. Numerous experimental studies have highlighted the substantial involvement of lincRNAs in the pathogenesis of cardiovascular diseases. Specific alterations in lncRNAs have been proposed as novel diagnostic and prognostic biomarkers for coronary artery disease. For instance, a large-scale study in the Japanese population identified an association between a long non-coding transcript named MIAT and myocardial infarction (MI). Furthermore, studies have identified other lncRNAs, such as Wisper and Carl, that are involved in acute myocardial infarction (AMI) and apoptosis-related processes.

The long intergenic non-coding RNA predicting cardiac remodeling (LIPCAR) has shown significant associations with left ventricular (LV) remodeling in AMI patients, leading to heart failure. Elevated levels of LIPCAR have been observed in AMI patients. LIPCAR has been found to be closely linked to chronic heart failure and severe CAD. Plasma levels of LIPCAR have emerged as valuable predictors of diastolic dysfunction in patients with type 2 diabetes. Other circulating lncRNAs, such as H19 and ANRIL, have also been associated with coronary artery disease and increased risk of chronic heart failure and stent restenosis. Therefore, the detection of lncRNAs in extracellular fluids represents a promising approach for non-invasive diagnosis and the prognostic assessment of coronary artery disease and other pathologies [106,107,108,109,110,111,112,113,114,115,116,117,118,119,120,121,122,123,124,125,126,127,128,129,130,131,132].

### 4.3. Circular RNA 

CircRNAs have emerged as an exciting field in molecular research, adding to the repertoire of non-coding RNAs. circRNAs are formed through a unique back-splicing mechanism that creates a stable circular structure lacking the usual 5′ cap and 3′ tail. Unlike linear RNAs, circRNAs are resistant to degradation by RNAse, making them highly stable. The discovery of circRNAs dates back to the 1970s when they were first observed in RNA viruses using electron microscopy. However, it was not until the advent of high-throughput RNA sequencing that their significance began to be recognized.

CircRNAs have garnered attention as potential biomarkers due to their involvement in various diseases, including CAD. They are ubiquitously present in eukaryotes, predominantly found in the cytoplasm. In different species ranging from fungi to mammals, circRNAs exhibit tissue-specific and developmental stage-specific expression patterns. They serve as versatile regulators of gene expression, participating in transcriptional and translational control, alternative splicing, and protein binding, and they act as sponges for microRNAs.

Recent research has shed light on the potential role of circRNAs in the pathogenesis of CAD. Their dysregulation has been linked to the initiation and progression of this cardiovascular condition. However, the field of circRNAs research is still in its early stages, and further studies are needed to explore their diagnostic and prognostic potential as cardiovascular disease biomarkers.

In summary, circRNAs have emerged as a promising area of research with implications in various diseases, including CAD. Understanding their functions and regulatory roles may provide valuable insights into disease mechanisms and pave the way for novel diagnostic and therapeutic approaches [133,134,135,136,137,138] (Table 3).

## 5. Biomarkers of CAD

Epigenetic biomarkers encompass various epigenetic mechanisms that provide valuable insights into disease pathology, facilitate disease detection, predict future disease risk, determine disease significance, monitor drug response, and guide therapy. Numerous epigenetic biomarkers have been identified for coronary artery disease (CAD) and its progression. These biomarkers can manifest as covalent modifications in DNA/histones or alterations in the expression patterns of non-coding RNAs (ncRNAs). Promisingly, specific gene methylation patterns hold potential as epigenetic biomarkers for CAD diagnosis and prognosis (Table 1). Extensive research has also elucidated the crucial role of miRNAs in the development and progression of CAD. Hence, miRNAs emerge as pivotal regulators of cellular gene expression during CAD onset and progression. Altered levels of various circulating miRNAs have shown promise as diagnostic and prognostic markers for CAD (Table 2). Furthermore, both lncRNAs and circRNAs have been implicated as potential biomarkers for CAD (Table 3), further expanding the repertoire of epigenetic biomarkers in this disease context [143,144,145].

Interleukin-6 (IL-6) is linked to an increased risk of cardiovascular events, specifically myocardial infarction. This is because IL-6 promotes the development of unstable atherosclerotic plaques or intensifies the inflammatory response of existing plaques. By elevating the levels of C-reactive protein, IL-6 contributes to a pro-inflammatory state, potentially affecting cardiac performance. The inflammatory burden caused by IL-6 can lead to oxidative stress, characterized by an increase in reactive oxygen species (ROS) production. This, in turn, can result in a reduced ejection fraction [146,147].

Reactive oxygen species have a significant impact on cellular functions, and maintaining a balanced redox state between ROS and antioxidants is crucial for cellular health. Disruptions in this redox balance lead to increased ROS production and oxidative damage. In response to ROS, several microRNAs (miRNAs) are expressed to help regulate oxidative stress. Conversely, oxidative stress can cause an upregulation of specific miRNAs that act as buffers against ROS-induced damage. Climent et al. highlighted the role of miRNAs in oxidative stress. They identified miR-34a, miR-144, miR-421, miR-129, miR-181c, miR-16, miR-31, miR-155, miR-21, and miR-1/206 as critical mediators. Furthermore, it has been recently discovered that miRNAs can serve as potential targets and modulators of pathways associated with oxidative stress, particularly involving nuclear factor erythroid 2-related factor 2 (NRF2), sirtuin (SIRT-1), and the NF-kB signaling cascade [143,144,145,146,147,148].

Yiqun G. et al. [149] revealed a significant influence of 5mC regulators on the diagnostic accuracy of distinguishing stable coronary artery disease (CAD) from acute myocardial infarction (AMI). We identified nine key 5mC regulators (DNMT3B, MBD3, UHRF1, UHRF2, NTHL1, SMUG1, ZBTB33, TET1, and TET3) that could serve as potential latent biomarkers in AMI. Furthermore, we identified two distinct clusters of 5mC molecular patterns and conducted an analysis of immune cell infiltration and pathway activity within each cluster. These findings open up new avenues for further research into the molecular mechanisms involving 5mC regulators in the progression from stable CAD to AMI. A diagnostic model was constructed using nine key 5mC regulators (DNMT3B, MBD3, UHRF1, UHRF2, NTHL1, SMUG1, ZBTB33, TET1, and TET3). The results of this model revealed the significant influence of these 5mC regulators and unveiled intriguing epigenetic insights when comparing the AMI population to those with stable CAD.

## 6. Epigenetic in CAD Patients

Contrary to the conventional belief, every individual’s genome is not identical. Extensive research has shown that significant variations exist in the epigenome, which is influenced by factors such as environmental exposure and pollutants. These epigenetic modifications contribute to the development of diseases like CAD. Recognizing this, scientists and medical professionals are striving to develop new therapeutic strategies based on these variations. The Furnishing the Human Genome project plays a pivotal role in understanding the intrinsic nature of genomics. With advancements in technology, such as next-generation sequencing, researchers have gained deeper insights into the role of candidate genes and their nucleotide sequence alterations in disease development. However, the field of epigenetics remains largely unexplored in the context of precision medicine for CAD. Nevertheless, researchers worldwide are already making efforts to unravel the epigenomic complexities associated with coronary artery disease. Various epigenome-based techniques have emerged to investigate the intricate world of epigenetics in CAD [150,151,152,153,154,155].

## 7. Potential Targets in CAD Therapy

The changes in environmental exposure can be viewed as an epigenetic indicator for potential treatment interventions. Scientists propose that these modifications can potentially be reversed through the use of pharmaceutical agents and nutraceuticals. For instance, extensive research has focused on the utilization of DNA methyltransferase inhibitors (DNMTis), histone acetyltransferase inhibitors, histone deacetylase inhibitors, histone methyltransferase inhibitors, and bromodomain and extra-terminal (BET) inhibitors as potential treatment options for inflammation and coronary artery disease (CAD). While natural DNMTs are found in certain food products, synthetic variants are also widely employed.

MiRNA-based treatments are expected to play a pivotal role in the future for preventing recurrences. However, it is essential to carefully assess the effectiveness of these drugs in relation to their off-target effects, which may include potential negative impacts on the complement system, vector-related toxicity, or interference with innate immunity. While some antagomiRs have been used in clinical studies for conditions such as hepatitis C virus, it is important to note that miRNA-targeted therapy is not without side effects.

As an illustration, blocking miR-320a could potentially lead to the downregulation of VEGF and other proliferation factors, potentially resulting in left ventricular dysfunction and a decreased ejection fraction. Other potential side effects encompass hypertension due to reduced nitric oxide synthesis and its associated effects, as well as thromboembolic events resulting from vascular endothelial damage caused by decreased angiogenic factors and increased platelet aggregation. A crucial consideration in utilizing miRNA as a therapeutic target is ensuring stability and protection against degradation enzymes. Current preclinical research is focused on employing nucleotides and antisense vectors that mimic miRNAs, thereby preventing their degradation or transcriptional blocking. Promising strategies to enhance stability include incorporating 2-O-methyl or 2-fluoro modifications and employing a 3′-cholesterol tail. Additionally, there is ongoing investigation into improving delivery systems to enhance kinetic parameters, with liposomes and microbubbles showing significant potential. These advancements may open up new therapeutic possibilities, particularly for addressing the hypofunctional myocardial tissues associated with conditions like ischemic heart disease or dilated cardiomyopathy [147,156,157,158,159,160,161,162].

## 8. Personalized Medicine

Healthcare providers have continually aimed to provide the highest quality care treatments to their patients, as previously discussed. They have explored a range of treatment strategies, identifying the most successful ones through careful observation, and shared their discoveries to build upon the methods of those who came before them. The ultimate goal has always been to offer accurate, tailored, and efficient treatments for each person, regardless of the constraints of the tools available at the time. Nonetheless, thanks to the advent of modern innovative techniques like genetic testing, advanced data analysis, the power of supercomputing, and electronic data storage, medical professionals and scientists have taken precision medicine to unparalleled levels. Precision medicine aims to manage diseases with the ultimate goal of complete cure. While human genome sequencing has indeed revolutionized the field of genetic medicine and laid the foundation for personalized medicine, it has become increasingly clear that our genetic material alone cannot comprehensively elucidate the intricacies of development and aging. Furthermore, our genes alone are inadequate for predicting susceptibility to a wide range of diseases. This is where epigenetics comes into play, offering answers to long-standing questions and shedding light on the underlying causes of life-threatening diseases. Innovations in epigenetic research, such as bisulfite sequencing, enable the accurate mapping and quantification of epigenetic marks, providing insights into disease phenotypes, including CAD, that were not possible with traditional genetic research techniques.

Accurately mapping and quantifying epigenetic markers improve clinical practices and early disease diagnosis even before genetic changes occur.

Research indicates that epigenetic variations often occur before genetic alterations in the development of diseases. This allows for clinical intervention before symptoms manifest, enhancing the treatment process and success rates. Epigenetic changes play a significant role in disease phenotypes such as heart diseases, neurological disorders, and cancer, offering crucial insights into their development and progression.

The concept of personalized medicine, particularly in the context of CAD, can significantly enhance disease management. Environmental factors, which influence epigenetic changes, are potential influencers of CAD. Therefore, characterizing epigenetic aberrations in precision CAD medicine requires careful consideration.

Overall, the combination of advanced techniques, genetic information, and epigenetic insights has propelled medicine into the realm of precision, enabling tailored approaches to disease management and improved patient outcomes [163,164,165,166,167,168].

## 9. Gap in Evidence

Even though there is a lot of research on epigenetics and its implications, we have not yet reached any definite conclusions. We know that epigenetic changes can alter the course of conditions such as CAD, but we still do not know for certain how these epigenetic alterations positively or negatively influence diseases. The majority of the literature talks about pathways, molecules, and so on, but a significant gap exists in terms of how to leverage this knowledge to our advantage. Of course, we are discussing a complex topic that is difficult to analyze from all perspectives. In our opinion, there is a need to concentrate unequivocally on the energies and knowledge in this field. We believe that personalized medicine will become the therapeutic standard in the future if this literature gap is filled.

## 10. Conclusions

This review provides a summary of recent discoveries in the field of epigenetics and their relevance to CAD. It highlights the significant impact of epigenetic mechanisms on our understanding of gene regulation in CAD, although the exact magnitude of this effect remains uncertain. The epigenome, which is highly responsive to environmental changes throughout an individual’s lifespan, plays a crucial role in regulating key biological processes and is closely associated with the expression of phenotypic traits and the risk of developing diseases. However, due to variations in environmental influences across different ethnic populations, it is challenging to generalize findings, making regional studies imperative. Moreover, there is still much to explore regarding the epigenetic aspects of in utero and postnatal exposures, including dietary factors. Moving forward, future research should focus on identifying potential diagnostic and therapeutic biomarkers for atherosclerotic disorders, with the aim of translating emerging epigenetic knowledge into practical applications for patient care. By harnessing the power of epigenetics and employing advanced techniques for studying epigenetic factors, healthcare professionals and researchers have the potential to advance personalized medicine to unprecedented levels compared to their predecessors. 

## Figures and Tables

**Table 1:** Methylation status in CAD.

Candidate Gene	Technique	Methylation Site	Methylation Pattern	Expression Pattern	Ref.
MTCT3	Bisulfite sequencing	Exon2	Hypermethylation	Downregulation	[49]
ES α	Nested methylation specific polymerase chainreaction (MS PCR)	Promoter	Hypermethylation	Not measured	[50]
GLANT2, HMGCR, CGK eNOS3	Targeted bisulfite sequencing	Promoter	Hypermethylation		[51]
LDLR	Targeted bisulfite sequencing	Promoter	Hypermethylation		[52]
Esβ	Methylation-specific PCR (MS PCR)	Promoter	Hypermethylation	Downregulation	[53]
IL6	Bisulfite treatment and pyrosequencing	Promoter	Hypermethylation	Not measured	[51]

**Table 2:** miRNA in CAD.

Reference	miRNAs	Findings
[94]	miRNA-155, miRNA-145, miRNA-17-a, miRNA-92-a, miRNA-133-a and miRNA-208-a	- Downregulated in patients CAD - Upregulated in patients CAD
[95]	miRNA-155, miRNA-145	- Shows significant downregulation in asymptomatic patient CAD compared to healthy subjects.
[96]	miRNA-133	- Exhibits significantly higher levels in acute myocardial infarction (AMI) when compared to the control group. It is also positively correlated with the severity of CAD
[97]	miRNA-370 and miRNA-208-a	- Both miRNAs exhibit notably elevated expression levels in individuals with coronary artery disease (CAD) when compared to the control group. - miRNA208a displays distinct expression patterns in heart muscle tissue. - miRNA370 is linked to lipid metabolism.
[98]	miRNA-208a	- Correlated with the severity of coronary artery disease (CAD).
[99]	miR-208, miRNA-499, miRNA-133 and miRNA-1	- Exhibited higher expression levels in patients with acute myocardial infarction (AMI) in comparison to healthy controls.
[100]	miRNA-340, miRNA-451, miRNA-624	- Demonstrated increased expression levels in patients with coronary artery disease (CAD) when compared to healthy controls.
[101]	miRNA-208-a	- Elevated in patients with acute myocardial infarction (AMI) in comparison to healthy individuals.
[102]	miRNA-499	- This biomarker has a higher sensitivity (5%) compared to troponin in diagnosing acute myocardial infarction (AMI). Additionally, its levels are increased in AMI patients when compared to congestive heart failure (HF) patients. This suggests that it may be a more reliable indicator for diagnosing AMI and distinguishing it from congestive heart failure.
[103]	miRNA-499-5-p	- This biomarker exhibits a stronger correlation with AMI patients who do not show ST elevation, particularly in aged patients, compared to troponin. In this context, it may serve as a more effective diagnostic marker for identifying AMI in elderly patients who do not display ST elevation on an electrocardiogram.
[104]	miRNA-133	- This biomarker shows significant upregulation in both human acute myocardial infarction (AMI) subjects and animal models of AMI. The increased expression of this biomarker in both clinical and experimental AMI settings suggests its potential importance in the pathophysiology and diagnosis of AMI.
[105]	miRNA-133-a	- This biomarker exhibits a significant increase in AMI (acute myocardial infarction). The elevated levels of this biomarker specifically in AMI patients may serve as a valuable diagnostic tool for distinguishing AMI from other coronary heart conditions.

**Table 3:** circRNA associated in CAD caption.

Ref.	Type of circRNA	Methods	Sample Source	Expression Pattern
[139]	Hsa_circ0124644 Hsa_circ0098964	Microarray Real-time q PCR	Blood	Upregulation
[140]	MIRCA	Real time q PCR	Blood	Downregolation
[141]	Hsa_circ11783	Microarray Real time q PCR	Blood	Downregolation
[142]	Hsa_circ000145	Real time q PCR	Plasma	Downregolation

## Data Availability

Not applicable.
